# Commentary: Cardiovascular risk according to body mass index in women of reproductive age with polycystic ovary syndrome: A systematic review and meta-analysis

**DOI:** 10.3389/fcvm.2022.920144

**Published:** 2022-08-10

**Authors:** Yani Ke, Kaihan Wu, Shan Liu

**Affiliations:** ^1^The Second Clinical Medical College of Zhejiang Chinese Medical University, Hangzhou, China; ^2^The First Clinical Medical College of Zhejiang Chinese Medical University, Hangzhou, China; ^3^Department of Clinical Evaluation Center, The First Affiliated Hospital of Zhejiang Chinese Medical University, Hangzhou, China

**Keywords:** polycystic ovary syndrome, cardiovascular risk, reproductive age, body mass index, meta-analysis

## Introduction

Recently, we read a systematic review and meta-analysis written by Zhuang et al. (1), which is of great clinical significance and value. It was found that high-baseline blood pressure and dyslipidemia were common in women of reproductive age with PCOS: mainly, the increase of SBP and DBP, TG, nonHDL-C, and LDL-C and the decrease of HDL-C. However, these changes seem to have nothing to do with BMI.

## Discussion

There were four retrieved databases mentioned in the abstract (the Cochrane Library, EMBASE, MEDLINE, and PubMed), but only three (the Cochrane Library, EMBASE, and MEDLINE) were demonstrated in the *Search Strategy*. The search strategy formats of PubMed and MEDLINE are different, but they are the same in this review, so the authors should clarify which one this strategy refers to. Meanwhile, the expression of medical subject headings in Pubmed-Medline (Table 1) sometimes used “mh” and sometimes used “Mesh.” It is recommended to use the same expression in one database. In addition, the search terms for different databases in the *Study Design* were not consistent. Adopting a proven and reliable search strategy is very necessary to obtain all the relative studies.

**Table 1:** Extracted information do not match in original review and in included article.

**No**	**Author**	**Indicator**	**In original review**	**In included article**
1	Adali et al. (2) Erdogan et al. (3) Ketel et al. (4) Long et al. (5) Luque-Ramirez et al. (6) Shroff et al. (7)	BMI	The BMI of PCOS group and control group should be matched.	No mention matching
2	Akram et al. (8)	Number of control group	50	30
		BMI	PCOS 23.3 ± 0.67 Control 21.8 ± 1.02	PCOS 23.6 ± 0.50 Control 23.5 ± 0.71
3	Adali et al. (2)	BMI	/	PCOS 24.40 ± 4.23 Control 23.90 ± 3.95
4	Alexandraki et al. (9)	BMI	PCOS 25.41 ± 0.80 Control 25.05 ± 1.19	PCOS 27.42 ± 1.12 Control 25.0 ± 1.19
		SBP	/	PCOS 114.81 ± 2.85 Control 111.6 ± 2.32
		DBP	/	PCOS 73.89 ± 2.25 Control 71.30 ± 1.70
5	Berneis et al. (10)	Number of two groups	PCOS 30 Control 24	PCOS 42 Control 37
		BMI	PCOS 28.4 ± 5.8 Control 28 ± 4.4	PCOS 27 ± 5 Control 26 ± 4
		Age	PCOS 25.1 ± 4.2 Control 25.5 ± 3	PCOS 28 ± 7 Control 31 ± 2
6	Kargili et al. (11)	TG	/	PCOS 90.9 ± 28.2 Control 89.0 ± 22.5
7	Ni et al. (12)	HDL	*Outcomes* include HDL-C	Not found
8	Shroff et al. (7)	NonHDL	*Outcomes* include nonHDL-C	Not obtained, no TC
9	Yildiz et al. (13)	Number of PCOS group	595	59

For the *Study Selection and Criteria* section, the inclusion and exclusion criteria were relatively clear. As mentioned in this article, the BMI of the PCOS group and control group should be matched, and their age should be roughly in one range. However, only parts of the included articles were explicitly BMI-matched; more details are shown in Table 1. Additionally, it is better to clarify the exact meaning of “roughly in one range.” Finally, there was a contradictive expression about language. The exclusion criteria mentioned articles published in languages other than English, but the authors declared they operated “without any language restriction” during retrieval in the *Search Strategy* section.

For the *Data Extraction* section, since nonHDL was not involved in any included articles, the authors pointed out that the nonHDL value is TC minus HDL. A detailed formula of its mean and deviation or relative references would make the results more reliable. For the *Quality Evaluation* section, the NOS scores were inconsistent with the description in *Risk of Bias and Quality Assessment*. Table 2 demonstrates the inconsistent descriptions. For the *Analysis Characteristics* section, the incorrectly extracted information is shown in Table 1. For the *Result* section, some inconsistent descriptions are listed in Table 2. Moreover, SBP and WHR lacked sensitivity analysis in the *Result* section.

**Table 2:** Inconsistent information in the original review.

**No**	**Section**	**Indicator**	**Quote A**	**Quote B**
1	Quality Evaluation and Risk of Bias and Quality Assessment	NOS scores	In Table 3: 6 studies scored 8 points, 15 studies scored 7 points, 11 studies scored 6 points and 6 studies scored 5 points.	In *Risk of Bias and Quality Assessment*: only 1 article with 7 points, 1 article with 6 points, 3 articles with 4 points and below.
2	Statistical Analysis and Blood Pressure	SBP	In Figure 6: Alexandraki et al. (9) was included	In Table 2: Alexandraki et al. (9) did not included SBP
			In Figure 6: Kargili et al. (11), Ketel et al. (4), Luque-Ramirez et al. (6) and Orio et al. (14) were included	In Table 2: Kargili et al. (11), Ketel et al. (4), Luque-Ramirez et al. (6) and Orio et al. (14) included SBP
3		DBP	In Figure 7: Alexandraki et al. (9) was included	In Table 2: Alexandraki et al. (9) did not include DBP
			In Figure 7: Kargili et al. (11), Luque-Ramirez et al. (6), Orio et al. (14) were not include	In Table 2: Kargili et al. (11), Luque-Ramirez et al. (6) and Orio et al. (14) included DBP

The *Discussion* section was relatively detailed and clear. However, according to the inclusion and exclusion criteria, there were some inappropriate articles included and some incomplete data. Furthermore, it is noted that during the discussion of lipid profiles, the change of HDL in different subgroups seems to be ignored.

This meta-analysis links PCOS, obesity, and cardiovascular risk factors, which have great clinical guiding value. However, due to some inappropriate information, an updated meta-analysis is needed to better draw conclusions and clarify the impact of BMI on cardiovascular risk factors in patients with PCOS with reliable methods. Additionally, more rigorous and standardized clinical research reports are an important premise for reasonable systematic reviews with meaningful conclusions.

## Author contributions

SL: design study, drafting the article, and making critical revisions. YK and KW: data collection, analysis, and drafting of the article. All authors contributed to the article and approved the submitted version.

## Conflict of interest

The authors declare that the research was conducted in the absence of any commercial or financial relationships that could be construed as a potential conflict of interest.

## Publisher's note

All claims expressed in this article are solely those of the authors and do not necessarily represent those of their affiliated organizations, or those of the publisher, the editors and the reviewers. Any product that may be evaluated in this article, or claim that may be made by its manufacturer, is not guaranteed or endorsed by the publisher.
